# Isolation of anticancer constituents from *Cucumis prophetarum* var. prophetarum through bioassay-guided fractionation

**DOI:** 10.1186/s12906-018-2295-5

**Published:** 2018-10-09

**Authors:** Abdulrhman Alsayari, Lucas Kopel, Mahmoud Salama Ahmed, Hesham S. M. Soliman, Sivakumar Annadurai, Fathi T. Halaweish

**Affiliations:** 1 Department of Pharmacognosy, College of Pharmacy, King Khalid University, Abha, Saudi Arabia; 2 Kalexsyn, 4502 Campus Drive, Kalamazoo, MI 49008 USA; 3 Department of Pharmaceutical Chemistry, Faculty of Pharmacy, The British University in Egypt, Al-Sherouk City, Cairo Egypt; 4 Department of Pharmacognosy, Helwan University, Cairo, Egypt; 5 Department of Chemistry and Biochemistry, South Dakota State University, Brookings, SD 57007 USA

**Keywords:** Bioassay-guided fractionation, Cucurbitacins, *Cucumis prophetarum* var. prophetarum, Anticancer, Breast cancer, Preliminary SAR

## Abstract

**Background:**

*Cucumis prophetarum* var. prophetarum is used in Saudi folk medicine for treating liver disorders and grows widely between Abha and Khamis Mushait City, Saudi Arabia.

**Methods:**

Bioassay-guided fractionation and purification were used to isolate the main active constituents of *Cucumis prophetarum* var. prophetarum fruits. These compounds were structurally elucidated using NMR spectroscopy, mass spectral analyses and x-ray crystallography. All fractions, sub-fractions and pure compounds were screened for their anticancer activity against six cancer cell lines.

**Results:**

The greatest cytotoxic activity was found to be in the ethyl acetate fraction, resulting in the isolation of five cucurbitacin compounds [E, B, D, F-25 acetate and Hexanorcucurbitacin D]. Among the cucurbitacins that were isolated and tested cucurbitacin B and E showed potent cytotoxicity activities against all six human cancer cell lines.

**Conclusion:**

Human breast cancer cell lines were found to be the most sensitive to cucurbitacins. Preliminary structure activity relationship (SAR) for cytotoxic activity of Cucurbitacins against human breast cancer cell line MDA-MB-231 has been reported.

## Background

The advances in natural product screening coupled with the growing appreciation for functional assays and phenotypic screens have contributed to the re-emergence of natural products for drug discovery in the genomics era [1]. Natural products have played a significant role in human disease therapy and compounds derived from natural products have always been noted as a valuable source for drug discovery [2]. Saudi flora contains 2250 species arranged in 142 families; among these, more than 1200 species are expected to be medicinal [3]. Several plant families in Saudi flora have been reported to have medicinal properties, such as the Cucurbitaceae family which is commonly used in Saudi folk medicine, a number of plant species from the Cucurbitaceae family, such as *Citrullus colocynthis*, have been utilized for the treatment of various health disorders [4–6].

Cucurbitacins are a group of highly oxygenated tetracyclic triterpenoids existing widely in the plant kingdom, especially in the Cucurbitaceae family [7]. A total of 12 classes of cucurbitacins have been recognized based on their structural characteristics and designated alphabetically from A to T with over 200 derivatives. Eight most active cucurbitacin components against cancer are cucurbitacin B, D, E, I, IIa, L glucoside, Q and R [8]. A number of cucurbitacins have been reported to be isolated from the genus Cucumis. Cucurbitacin (B, E, I, O, P and Q1); dihydrocucurbitacin (D and E), isocucurbitacin (B, D and E) and dihydroisocucurbitacin (D and E) have been reported to be isolated from *Cucumis prophetarum* L. Cucurbitacin B and Dihydrocucurbitacin B isolated from *Cucumis prophetarum* L., were studied for their cytotoxic activity towards human cancer cell lines, mouse embryonic fibroblast (NIH3T3) and virally transformed form (KA3IT) cells [9]. Recently the antidiabetic and antioxidant activity of the different fractions of fruits of *Cucumis prophetarum* L. has been reported [10].

*Cucumis prophetarum* var. prophetarum (Cucurbitaceae), which is locally called as Shari-al-deeb, is used in Saudi folk medicine for the treatment of liver disorders and grows widely between Abha and Khamis Mushait City, Saudi Arabia. To the best of our knowledge there are no studies reported on this variety.

The aim of the present study was the extraction, isolation, and structural elucidation of the active constituents with potential anti-cancer activity from *Cucumis prophetarum* var. prophetarum using bioassay-guided fractionation. The anticancer activities of the extracts, fractions, and pure isolated compounds obtained from the bioassay-guided fractionation were evaluated in vitro using six human cancer cell lines: breast (MCF7, MDA-MB-231), colon (HCT-116), ovarian (A2780/ A2780CP), and liver (HepG2). The chemical structures of the pure isolated compounds were elucidated using NMR spectroscopy, mass spectral analyses, and x-ray crystallography.

## Methods

^1^H-NMR, ^13^C-NMR, and 2D-NMR were conducted using Bruker AVANCE-400 MHz and 600 MHz NMR spectrometers at 22 °C, in deuterated chloroform (CDCl_3_) using tetramethylsilane (TMS) as the internal standard; chemical shifts are given in ä (ppm) values. High-resolution ESI mass spectra were measured on a ThermoFinnigan MAT 95 XL mass spectrometer at the mass spectroscopy facility located at the University of Buffalo (Buffalo, NY, USA). X-ray crystal structure was obtained with a Bruker-AXS Photon-100 diffractometer at the X-Ray Crystallographic Laboratory, University of Minnesota (Minneapolis, MN, USA). Column chromatography was carried out using silica gel (230–400 mesh) purchased from Sorbent Technologies (Norcross, GA, USA). TLC was performed using pre-coated silica gel PE Sheets purchased from Sorbent Technologies (Norcross, GA, USA), visualized under ultraviolet at 254 nm, and stained with Ceric Ammonium Molybdate (CAM) followed by heating. All solvents were obtained from commercial suppliers and used as received.

Dimethyl sulfoxide (DMSO), 3-(4,5-Dimethyl-2-thiazolyl)-2,5-diphenyl-2H-tetrazolium bromide (MTT), sodium dodecyl sulfate (SDS), and RPMI 1640 medium were purchased from Sigma-Aldrich (St. Louis, MO, USA). Dulbecco’s modified Eagle medium (DMEM), antibiotics, phosphate buffered saline (PBS) 1X solution, and trypsin were purchased from Gibco (Grand Island, NY, USA). Fetal bovine serum (FBS) was purchased from HyClone (Logan, UT, USA).

### Plant materials

Fresh fruits of *Cucumis prophetarum* var. prophetarum were collected in June 2010 from the wild near Abha-Khamis Road, Abha, Saudi Arabia. The plant was botanically authenticated and a voucher specimen was deposited in the Pharmacognosy Department Herbarium, College of Pharmacy, King Khalid University, Abha, Saudi Arabia.

### Preparation of plant extracts and fractions

The fruits of *Cucumis prophetarum* var. prophetarum (6.5 kg) were cut into pieces and homogenized in methanol (a blender was filled to 1/3 volume with fruits, 1.5 L of methanol was added, then the mixture was homogenized for 5 min). The mixture was then macerated in methanol for a further 72 h. The methanol extract was filtered, concentrated under reduced pressure at 40 °C using a rotary evaporator, and lyophilized to afford a residue (200 g, 3.07%). The dried methanol extract (160 g) was divided into several portions of 20 g and each of them was dispersed in de-ionized water (500 ml) and partitioned sequentially with n-hexane (500 ml × 3), ethyl acetate (500 ml × 3), and n-butanol (500 ml × 3). The combined solvent of each partitioned extract was concentrated under reduced pressure at 40 °C using the rotary evaporator and freeze dried for 72 h to yield an n-hexane fraction (2.5 g, 0.03%), an ethyl acetate fraction (4.5 g, 0.07%), n-butanol (4.5 g, 0.07%), and the remainder of the water fraction (91 g, 1.40%). All fractions were dissolved in DMSO, with the exception of the water fraction which was dissolved in media, and they were tested for their anti-cancer activities using six human cancer cell lines [11].

### Isolation

According to the bioassay-guided fractionation, the ethyl acetate fraction showed the greatest anti-cancer activity, and thus was selected for the present study (Table 4). The EtOAc fraction was subjected to column chromatography on silica gel (300 g) and eluted with stepwise gradients of n-hexane/EtOAc (100:0, 90:10, 80:20, 70:30, 60:40, 50:50, 45:55, 40:60, 30:70, 20:80, 10:90, 0:100 *v*/v) and finally with 2 L methanol. A total of 475 fractions (25 mL each) were collected and combined on the basis of their TLC profiles into three main fractions as follows: fraction I (1–186) (766.8 mg, 0.011%), Fraction II (187–226) (655 mg, 0.010%), and Fraction III (227–475) (1.206 g, 0.018%).

Fraction I (306 mg) was subjected to preparative TLC using (n-hexane/EtOAc, 7:3) to yield band 1 (a mixture of compound 1 and 2) (15.8 mg, 0.00024%) and band 2 (pure compound 2) (35.3 mg, 0.00054%). Fraction II crystallized (on standing) yielding compound 2 (655 mg, 0.010%). Fraction III (933.7 mg) was chromatographed again on a silica gel (80 g) and eluted with dichloromethane/ methanol (100:0, 98:2). A total number of 154 subfractions (10 mL each) were collected and combined on the basis of their TLC profiles into three main subfractions, as follows: subfraction A (1–72) (222.25 mg, 0.0034%), subfraction B (73–97) (423.8 mg, 0.0065%), and subfraction C (98–15) (79.9 mg, 0.0012%).

Subfraction A yielded compound 3 (106.6 mg, 0.0016%), subfraction B yielded compound 4 (99.8 mg, 0.0015%), and subfraction C yielded compound 5 (51.6 mg, 0.00079%).

### Cell cultures

Human cancer breast cell lines (MCF7, MDA-MB-231), human cancer colon cell lines (HCT-116), human ovarian carcinoma cell lines (A2780/ A2780CP), and human liver carcinoma cell lines (HepG2) were obtained from American Type Cell Culture (ATCC, Rockville, MD, USA). The MDA-MB-231, A2780, and A2780CP cell lines were maintained at 37 °C in a humidified atmosphere of 5% CO_2_ in RPMI-1640 medium containing 10% fetal bovine serum and antibiotics (100 IU/mL penicillin and 100 μg/mL streptomycin). The HepG2, HCT-116, and MCF7 cell lines were maintained at 37°C in a humidified atmosphere of 5% CO_2_ in a DMEM medium containing 10% fetal bovine serum and antibiotics (100 IU/mL penicillin and 100 μg/mL streptomycin).

### MTT assay

The effects of all fractions and pure compounds were tested on six human cancer cell lines (MCF7, MDA-MB-231, HCT-116, A2780, A2780CP and HepG2) using a 3-(4,5-dimethylthiazol-2-yl)-2,5-diphenyltetrazolium bromide (MTT) assay, which measures the ability of metabolically active cells to convert tetrazolium salt into a blue formazan product. Cells (1 × 10^4^cells/well) were seeded into a 96-well plate and allowed to attach to the well over night. All plant fractions or pure compounds were dissolved in DMSO at10 mM and then diluted in culture medium (The final DMSO concentration did not exceed 1%). Plant fractions or pure compounds were added at different concentrations (0, 1.6, 8, 40, 200, 1000 μg/ml for each fraction and 0, 0.16, 0.8, 4, 20, 100 μM for pure compounds) and cells were incubated for a further 48 h. After incubation, 10 μL of 5 mg/mL of MTT dye were added to the cells for 4 h at 37 °C, followed by the addition of 100 μL of 10% SDS in 0.01 N HCl as a solubilizing agent. The absorbance at 570 nm was recorded using an ELISA microplate reader. The results of viability were expressed as a percentage of the control and IC_50_ concentrations with 50% growth inhibitory effects were calculated from a dose–response curve.

## Results

### Isolation and structural elucidation

**Fig. 1:**
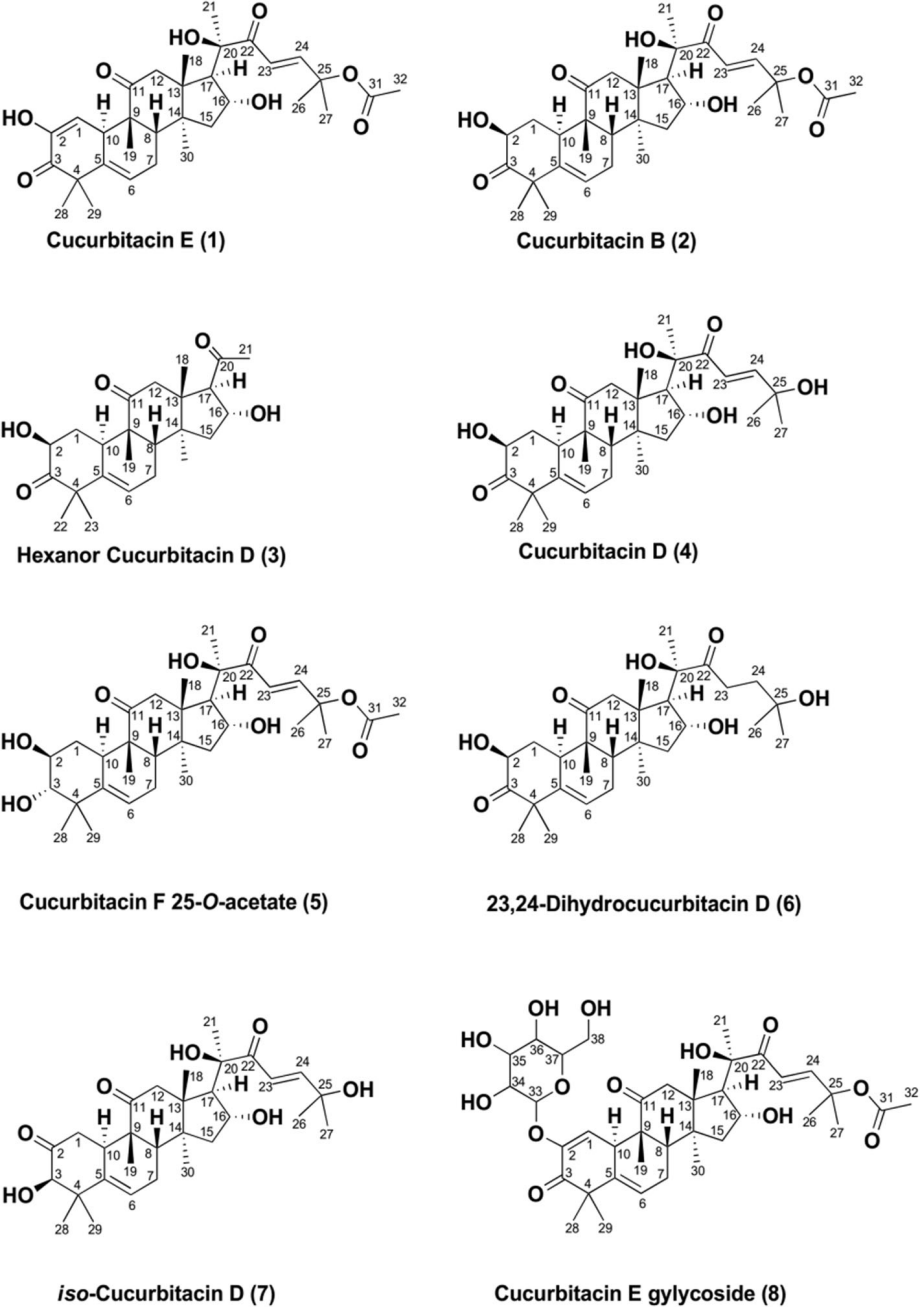
Chemical Structures of Isolated Cucurbitacins

The methanolic extract of the fruits of *Cucumis prophetarum* var. prophetarum was dispersed in deionized water and partitioned sequentially with n-hexane, ethyl acetate, and n-butanol. Based on the bioassay-guided fractionation, the ethyl acetate fraction showed higher anticancer activity and thus it was subjected to a series of chromatography techniques to yield five Cucurbitacin compounds (Fig. 1).

Compound **2** was isolated as a white powder. It showed a molecular ion peak at m/z 581.30697 [M + Na]^+^ (calcd. 581.30849) in the HR-ESIMS spectrum, which corresponded to the molecular formula C_32_H_46_O_8_. The ^1^H- NMR spectral data of **2 (**Table 1**)** exhibited nine tertiary methyl group signals at δ_H_ 0.99 (3H, *s*, H-18); 1.08(3H, *s*, H-19); 1.29 (3H, *s*, H-28); 1.35(3H, *s*, H-29); 1.36 (3H, *s*, H-30); 1.43 (3H, *s*, H-21); 1.55 (3H, *s*, H-26); 1.57 (3H, *s*, H-27); 2.02 (3H, *s*, OAc), an olefinic proton at δ_H_ 5.80 (1H, *d*, *J* = 5.60 Hz, H-6), two *trans*-coupled olefinic protons on a side chain at δ_H_ 6.48 (1H, *d*, *J* = 15.6 Hz, H-23) and 7.07 (1H, *d*, *J* = 15.6 Hz, H-24), two hydroxymethine protons at δ_H_ 4.43 (1H, *dd*, *J* = 4.4, 12.9 Hz, H-2) and 4.36 (1H, *m*, H-16), and a pair of doublets at δ_H_ 2.69 (1H, *d*, *J* = 14.7 Hz, H-12β) and 3.25 (1H, *d*, *J* = 14.4 Hz, H-12α). The ^13^C-NMR spectral data of compound **2** revealed the presence of 30 carbon signals for a triterpene skeleton, in addition to two carbon signals for an acetate moiety. The ^13^C-NMR data **(**Table 2**)** showed nine methyl signals at δ_C_ 18.8, 19.8, 20.0, 21.2, 23.9, 25.9, 26.3, 29.3, 21.9 were assigned for C -30, C-18, C-19, C-29, C-21,C-27, C-26, C-28 and CH_3_CO, respectively, three carbonyls at δ_C_ 202.5, 212.3213.6 were assigned for C-22, C-11, C-2,respectively, four olefinic signals at δ_C_ 120.3, 120.4, 140.2, 151.9 were assigned for C-23, C-6, C-5, C-24, respectively, and four oxygenated functions at δ_C_ 71_._2, 71.6, 78.2, 79.3 were assigned for C-16, C-2, C-20 and C-25. The presence of a singlet methyl signal at δ_H_ 2.02 in ^1^H NMR spectra and two carbon signals at 21.9 and 170.3 in ^13^C NMR spectra indicated the presence of an acetate moiety at C-25. The above data indicated the presence of a cucurbitacin tetracyclic triterpene skeleton; a comparison of the data with those published [10, 12–14] indicated that structure **2** was characterized as cucurbitacin B. Further confirmation of compound **2** was achieved by comparison with an authentic sample of cucurbitacin B from our lab.

**Table 1:** ^1^H-NMR spectral data for compounds 2, 3, 4 and 5 in CDCl_3_^a^ (400 MHz)

H	(2)	(3)	(4)	(5)
1 α	2.32 ddd (3.3/ 5.8 /12.5)	2.24 m	2.33 ddd (3.3/ 5.8 /12.5)	s.o.
1β	1.21 d (13.0)	1.21 d (12.8)	1.21 m	1.15 d (6.30)
2	4.43 dd (4.44/12.9)	4.43 dd (6.0/12.8)	4.46 dd (6.5/12.8)	3.59 m
3	–	–	–	2.98 d (9.0)
4	–	–	–	–
5	–	–	–	–
6	5.80 d(5.6)	5.78 d br (5.6)	5.79 br m	5.73 d (5.4)
7α	s.o.	s.o.	1.94 m	s.o
7β	2.41 dm	2.41 dd (7.5/19.1)	2.40 dd (8.2/19.8)	2.39 m
8	1.98 br d (7.8)	2.01 d (6.8)	1.97 d br (7.9)	1.93 br d (7.6)
9	–	–	–	–
10	2.75 br d (13.1)	2.50 d (14.3)	2.78 d (13.7)	2.62 br d (14.4)
11	–	–		–
12α	3.25 d (14.4)	3.32 d (14.5)	3.32 d (14.3)	3.18 d (14.4)
12β	2.69 d (14.7)	2.76 d (12.7)	2.7 d (14.6)	2.52 d (6.81)
13	–	–	–	–
14	–	–	–	–
15α	1.88 dd(9.4/13.5)	s.o	s.o.	s.o.
15β	1.45 d (5.8)	1.93 dd (11.7/19.8)	1.84 dd (8.2/13.1)	1.85 m
16	4.36 m	4.92 m	4.33 m br	4.33 m
17	2.51 d (7.3)	3.17 d (6.45)	2.55 d (6.88)	2.48 d (7.03)
18	0.99 s	0.66 s	0.98 s	0.96 s
19	1.08 s	1.05 s	1.8 s	1.27 s
20	–	–	–	–
21	1.43 s	2.16 s	1.39 s	1.55 s
22	–	–		–
23	6.48 d (15.6)	–	6.60 d (15.1)	6.46 d (15.6)
24	7.07 d (15.6)	–	7.14 d (15.1)	7.07 d (15.6)
25	–	–	–	–
26	1.55 s	–	1.35 s	1.57 s
27	1.57 s	–	1.35 s	1.55
28	1.29 s	1.27 s	1.30 s	1.27 s
29	1.35 s	1.33 s	1.33 s	1.20 s
30	1.36 s	1.37 s	1.34 s	1.10 s
O_2_CMe	2.02 s	–	–	2.02 s

^a^ *j* values in Hz are given in parentheses, (so) signal obscured, (s) singlet, (d) doublet, (dd) doublet of doublets, (m) multiplet, (br) broad

**Table 2:** ^13^C-NMR spectral data for compounds 2, 3, 4 and 5 in CDCl_3_

C	(2^)a^	(3)^a^	(4)^a^	(5) ^b^
1	36.0	35.9	35.9	34.0
2	71.6	71.5	71.6	71.4
3	213.6	212.9	213.1	81.1
4	50.2	50.2	50.2	50.8
5	140.2	140.3	140.3	140.7
6	120.4	120.1	120.2	120.5
7	23.8	23.9	23.8	24.1
8	42.3	42.7	42.3	42.7
9	48.4	48.6	48.3	48.4
10	33.7	33.6	33.7	33.4
11	212.3	211.1	212.3	213.1
12	48.6	49.8	48.6	48.7
13	50.6	48.9	50.7	51.7
14	48.1	44.9	48.2	48.1
15	45.3		45.4	45.4
16	71.2	71.4	71.3	71.1
17	58.1	67.5	57.3	58.2
18	19.8	19.7	19.9	19.9
19	20.0	19.9	20.1	20.4
20	78.2	208.2	78.1	78.4
21	23.9	31.5	23.9	24.7
22	202.5	–	202.6	202.6
23	120.3	–	118.9	119.4
24	151.9	–	155.7	152.0
25	79.3	–	71.1	79.4
26	26.3	–	29.5	26.5
27	25.9	–	29.3	26.1
28	29.3	21.2	28.9	21.7
29	21.2	29.3	21.2	23.8
30	18.8	19.9	19.2	19.1
CH_3_COO	170.3, 21.9			170.4, 21.6

^a^ Measured at 100 MHz. ^b^ Measured at 150 MHz

Band **1** was obtained as a white amorphous powder and displayed two molecular ion peaks at m/z 579.29530 [M + Na]^+^ (calcd. 579.29284) and m/z 581.30967 [M + Na]^+^ (calcd. 581.30849) in its HR-ESIMS, corresponding to the molecular formulas C_32_H_44_O_8_ and C_32_H_46_O_8_, respectively. A comparison of the ^1^H-NMR spectrum of 1 with published data [15–17] led us to characterize band **1** as a mixture of cucurbitacin E (**1**) and cucurbitacin B (**2**).

Compound **4** was isolated as a yellow amorphous powder. It showed a molecular ion peak at m/z 539.29854 [M + Na]^+^ (calcd. 539.29792) in the HR-ESI-MS spectrum, which corresponded to the molecular formula C_30_H_44_O_7_. The ^1^H NMR spectral data of **4** (Table 1) exhibited eight tertiary methyl group signals at δ_H_ 0.98 (3H, *s*, H-18); 1.30 (3H, *s*, H-28); 1.33 (3H, *s*, H-29); 1.34 (3H, *s*, H-30); 1.35 (3H, *s*, H-26); 1.35 (3H, *s*, H-27); 1.39 (3H, *s*, H-21); 1.8 (3H, *s*, H-19), an olefinic proton at δ_H_ 5.79 (1H, *m*, *J* = 5.60 Hz, H-6), two *trans*-coupled olefinic protons on a side chain at δ_H_ 6.60 (1H, *d*, *J* = 15.16 Hz, H-23) and 7.14 (1H, *d*, *J* = 15.17 Hz, H-24), two hydroxymethine protons at δ_H_ 4.46 (1H, *dd*, *J* = 6.5, 12.8 Hz, H-2) and 4.33 (1H, *m*, H-16), and a pair of doublets at δ_H_ 2.7 (1H, *d*, *J* = 14.6 Hz, H-12β) and 3.32 (1H, *d*, *J* = 14.3 Hz, H-12α). The ^13^C-NMR spectral data of compound **4** revealed the presence of 30 carbon signals for a triterpene skeleton. The ^13^C-NMR data (Table 2) showed eight methyl signals at δ_C_ 19.2, 19.9, 20.1, 21.2, 23.9, 28.9, 26.3, 29.3, 29.5 which were assigned for C-30, C-18, C-19, C-29, C-21, C-28, C-27 and C-26, respectively; three carbonyls at δ_C_ 202.6, 212.3213.1 which were assigned for C-22, C-11 and C-3, respectively: four olefinic signals at δ_C_ 118.9, 120.2, 140.3, 155.7 which were assigned for C-23, C-6, C-5 and C-24, respectively, and four oxygenated functions at δ_C_ 71_._3, 71.6, 78.1, and 71.1 which were assigned for C-16, C-2, C-20 and C-25, respectively.

The above ^1^H- and ^13^C-NMR data of Compound **4** were similar to that of Compound **2**, except for the absence of a singlet methyl signal at δ_H_ 2.02 in the ^1^H-NMR spectrum and two carbon signals at 21.9 and 170.3 in the ^13^C-NMR spectra, indicating that the acetate group at C-25 of **2** was replaced by a proton in compound **4**. Thus, on the basis of spectral data and published data [18] compound **4** was defined as cucurbitacin D. Further confirmation of compound **4** was achieved by comparison with an authentic sample of cucurbitacin D in our lab.

Compound **3** was isolated as a yellow amorphous powder. It showed a molecular ion peak at m/z 425.23082 [M + Na]^+^ (calcd. 525.22985) in the HR-ESI-MS spectrum, which corresponded to the molecular formula C_24_H_34_O_5_. The ^1^H NMR spectral data of **3** (Table 1) suggested that the chemical shift of rings of A, B, and C were in agreement with those of compound **4**. However, the shifts of the two trans-coupled olefinic protons of the side chain were not detected and only five tertiary methyl group signals were found at δ_H_ 0.66 (3H, *s*, H-18), 1.05 (3H, *s*, H-19), 1.27 (3H, *s*, H-28), 1.33 (3H, *s*, H-29), and 1.37 (3H, *s*, H-30), in addition to a new tertiary methyl group signal at δ_H_ 2.16 (3H, methyl ketone). This is somewhat different than the eight tertiary methyl signals in compound **4**. The ^13^C-NMR data (Table 2) revealed the presence of 24 carbon signals, including six methyl signals at δ_C_ 19.7, 19.9, 19.9, 21.2, 29.3, 31.5 which were assigned for C-18, C-19, C-30, C-28, C-29, and C-21, respectively; two carbonyls at δ_C_ 211.1, 212.9 which were assigned for C-11 and C-3, respectively; two olefinic signals at δ_C_ 120.1, 140.3 which were assigned for C-6 and C-5, respectively, and two oxygenated functions at δ_C_ 71_._4 and 71.5 which were assigned for C-16 and C-2, respectively. This suggests a hexanorcucurbitacin skeleton [19]. As evident from the mass spectra, 114 amu differences were observed between the molecular ion peak of compound **3** (m/z 425) and that of compound **4** (m/z 539), indicating the loss of a side chain by the cleavage between C-20 and C-22 and the formation of a methyl ketone at C-21. On the basis of the above spectral data, along with reported ^13^C-NMR data in the literature [20] compound **3** was identified as hexanorcucurbitacin D.

Compound **5** was obtained as a white amorphous powder and displayed a molecular ion peak at m/z 583.32388 [M + Na]^+^ (calcd. 583.32414) in its HR-ESI-MS, corresponding to the formula C_32_H_48_O_8_. The ^1^H-NMR spectral data of compound **5** (Table 1) showed nine tertiary methyl group signals at δ_H_ 0.96 (3H, *s*, H-18), 1.10 (3H, *s*, H-30), 1.20 (3H, *s*, H-29), 1.27 (3H, *s*, H-28), 1.27 (3H, *s*, H-19), 1.55 (3H, *s*, H-21), 1.57 (3H, *s*, H-26), 1.55 (3H, *s*, H-27), and 2.02(3H, *s*, OAc), while resonances at δ_H_ 2.98 (1H, *d*, *J* = 9.0 Hz, H-3), 3.59 (1H, *m*, H-2), and 4.33 (1H, *m*, H-16) were assigned to proton signals attached to three oxygenated methine carbons. An olefinic proton at δ_H_ 5.73 (1H, *d*, *J* = 5.49 Hz, H-6) and two *trans*-coupled olefinic protons on the side chain at δ_H_ 6.46 (1H, *d*, *J* = 15.6 Hz, H-23) and 7.07 (1H, *d*, *J* = 15.6 Hz, H-24) were observed in the ^1^H NMR spectrum. In addition, a pair of coupled doublet protons was recognized at δ_H_ 2.52 (1H, *d*, *J* = 6.81 Hz, H-12β) and 3.18 (1H, *d*, *J* = 14.4 Hz, H-12α). The ^13^C-NMR spectrum of **5** displayed 32 carbon signals, of which 30 carbon signals were attributed to the triterpene skeleton and two carbon signals for an acetate moiety. As evident from the DEPT experiment, the ^13^C-NMR data (Table 2) showed nine tertiary methyl signals at δ_C_ 19.1, 19.9, 20.4, 21.7, 23.8, 24.7, 26.1, 26.5, and 21.6 which were assigned for C -30, C-18, C-19, C-28, C-29, C-21, C-27, C-26, and CH_3_CO, respectively; two carbonyls at δ_C_ 202.6 and 213.1 which were assigned for C-22 and C-11, respectively; four olefinic signals at δ_C_ 119.4, 120.5, 140.7 and 152.0 which were assigned for C-23, C-6, C-5, and C-24, respectively; and five oxygenated functions at δ_C_ 71_._1, 71.4, 78.4, 79.3, and 81.1 which were assigned for C-16,C-2,C-20,C-25 and C-3, respectively. The presence of a singlet methyl signal at δ_H_ 2.02 in the ^1^H-NMR spectra and two carbon signals at 21.6 and 170.4 in the ^13^C-NMR spectra indicated the presence of an acetate moiety at C-25. A comparison of the ^1^H- and ^13^C-NMR spectroscopic data between **5** and **2** showed similarities, although compound **5** exhibits the absence of a carbonyl signal and the presence of a new oxygenated carbon signal, suggesting the carbonyl in **2** was replaced by a hydroxyl group in **5**. This assumption was also supported by the analyses of the two-dimensional NMR spectrum (Table 3).

**Table 3:** NMR spectroscopic data of compound 5

NO	^13^C/ppm^a^	1H/ppm^b^ multiplicities (J/Hz)	HMBC
1	34.0	s.o., 1.15 d (6.30)	C-2, C-19, C-10, C-3,C-5,C-8,C-9
2	71.4	3.59 m	C-1, C-3,C-4,C-10
3	81.1	2.98 d (9.08)	C-28,C-29,C-1
4	50.8	–	C-28,C-29,C-6
5	140.7	–	C-7,C-1,C-10
6	120.5	5.73 d (5.49)	C-4,C-5,C-7,C-10,C-8
7	24.1	s.o., 2.39 m	C-6,C-8
8	42.7	1.93 br d (7.66)	C-30,C-19,C-7,C-15,C-6,C-10
9	48.4	–	C-19,C-12,C-10,C-8
10	33.4	2.62 br d (14.43)	C-6,C-8,C-19,C-1
11	213.1	–	C-12,C-19
12	48.7	3.18 d (14.49), 2.52 d (6.81)	C-18, C-17, C-11, C-13
13	51.7	–	C-12, C-15, C-17, C-18, C-30
14	48.1	–	C-30, C-18, C-12, C-7, C-16,C-8, C-15
15	45.4	s.o., 1.85 m	C-30, C-8
16	71.1	4.33 m	C-17, C-15
17	58.2	2.48 d (7.03)	C-18, C-21, C-12, C-16
18	19.9	0.96 s	C-12, C-13, C-14, C-17
19	20.4	1.27 s	C-10, C-8
20	78.4	–	C-21, C-16, C-17
21	24.7	1.55 s	–
22	202.6	–	C-24, C-23
23	119.4	6.46 d (15.6)	C-24
24	152.0	7.07 d (15.6)	C-27, C-26, C-23
25	79.4	–	C27, C26, C23
26	26.5	1.57 s	C-27, C-24
27	26.1	1.55 s	C-26
28	21.7	1.27 s	C-29
29	23.8	1.20 s	C-28
30	19.1	1.10 s	C-15, C-21, C-8

^a^Measured at 150 MHz, ^b^ Measured at 400 MHz

**Fig. 2:**
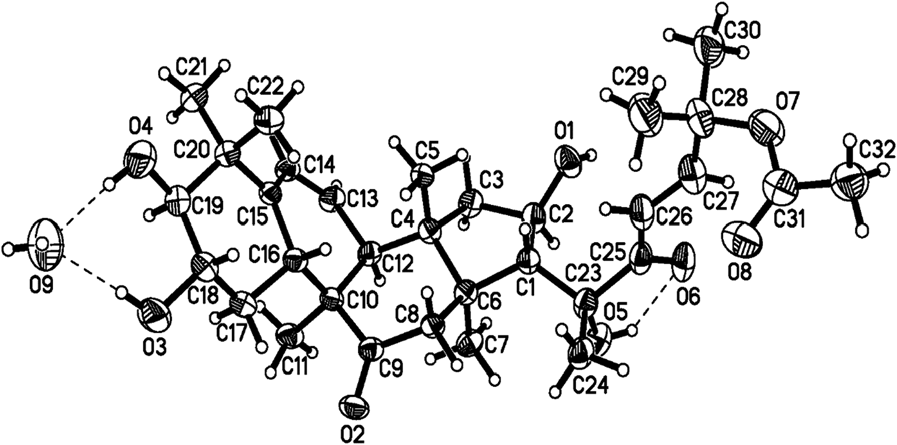
ORTEP representation of Compound 5

In the ^1^H-^1^H COSY spectrum, the methine proton at δ_H_ 2.98 (1H, *d*, *J* = 9.0 Hz, H-3) correlated with a methine proton at δ_H_ 3.59 (1H, *m*, H-2) while the HMQC spectrum showed a correlation between a methine proton at δ_H_ 2.98 (1H, *d*, *J* = 9.0 Hz, H-3) and an oxygenated carbon at δ_C_ 81.1 (C-3), as well as between a methine proton at δ_H_ 3.59 (1H, *m*, H-2) and an oxygenated carbon at δ_C_ 71.1, (C-2). Further confirmation for the proposed structure was obtained by X-ray single crystal (Fig. 2). Therefore, on the basis of above spectral evidence, the structure of 5 was identified as Cucurbitacin F 25 *O-*acetate.

### Biological evaluation

**Fig. 3:**
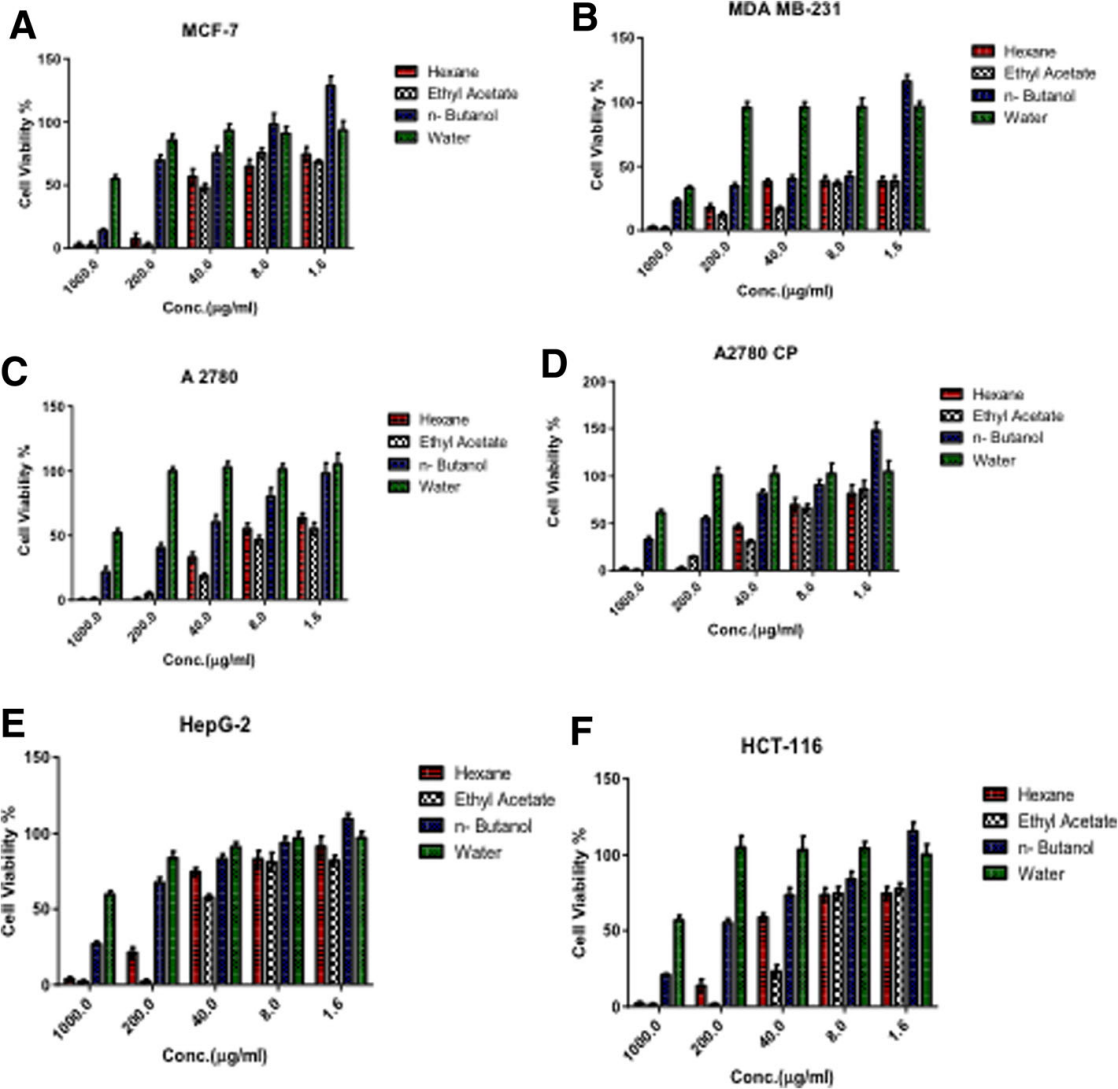
Inhibitory effects of n-hexane, ethyl acetate, n-butanol and aqueous fractions on proliferation of cancer cells (**a**-MCF-7; **b**-MDA-MB-231; **c**-A2780; **d**-A2780CP; **e**-HepG2 and **f**-HCT-116). Cells were treated with 0-1000 µg/ml of each fraction. MTT assay was used to measure the cell viability % after 48 hrs of treatment. The error bars indicate SD of *n* = 8 per concentration

**Fig. 4:**
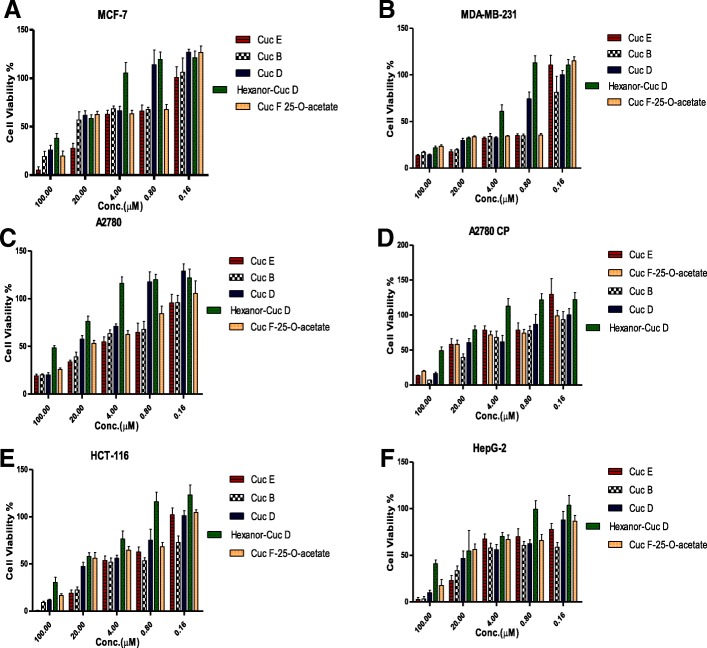
Inhibitory effects of Cucurbitacins **e**, **b**, **d**, **f** 25-O-acetate and hexanorcucurbitacin D on proliferation of cancer cells (**a**-MCF-7; **b**-MDA-MB-231; **c**-A2780; **d**-A2780CP; **e**-HCT-116 and **f**-HepG2). Cells were treated with 0-100 µM of each compound. MTT assay was used to measure the cell viability % after 48 hrs of treatment. The error bars indicate SD of *n* = 8 per concentration

The potential effects of the n-hexane, ethyl acetate, n-butanol and aqueous extracts and fractions (I and III) from the fruits of *Cucumis prophetarum* var. prophetarum on the proliferation of MCF7, MDA-MB-231, HCT-116, A2780, A2780CP, and HepG2 were investigated using the MTT assay for 48 h. Cell viability was measured in the concentration range of 0 μg/mL to 1000 μg/mL for each fraction (Fig. 3) and 0 μM to 100 μM for each pure compound (Fig. 4). As shown in (Table 4) the ethyl acetate fraction exhibits potential cytotoxic effects on the MCF-7, MDA MB-231, A2780, A2780 CP, and HCT-116 cell lines with IC_50_ 17.5, 0.35, 2.82, 19.2, and 14.2 μg/mL, respectively, while the n-hexane fraction was found to be active against the MCF-7, MDA MB-231, and A2780 cell lines with IC_50_ 19.7, 0.76, and 7.15 μg/mL, respectively. The n-butanol fractions demonstrated very weak cytotoxic activity against all cell lines, with IC_50_ values ranging from 43 to 358 μg /mL, whereas the water fraction showed no cytotoxic activity against any of the tested cell lines (> 1000 μg /mL). In addition, the ethyl acetate fraction showed a concentration-dependent inhibitory effect in the MCF-7, MDA MB-231, A2780, A2780 CP, and HCT-116 cell lines at ≥ 8 μg /mL, as did the n-hexane fraction suggesting that the ethyl acetate fraction possesses the highest cytotoxicity and led us to carry out a study to determine the active constituents that may be potential anticancer compounds.

**Table 4:** Cytotoxic effects of the tested fractions

	IC_50_^a^ (μg/mL)					
Fractions	MCF-7	MDA MB 231	A2780	A2780 CP	HepG2	HCT-116
Hexane fraction	19.7	0.76	7.15	20.27	55.4	25
Ethyl acetate fraction	17.5	0.35	2.82	19.2	28.5	14.2
n-Butanol fraction	218.4	43	94.01	273	358.5	169.5
Water fraction	> 1000	> 1000	> 1000	> 1000	> 1000	> 1000
Fraction I	3.5	0.15	4.9	5.9	0.27	0.15
Fraction III	6.80	0.12	20.5	17.5	0.52	0.65

Inhibitory effects of the fractions from the extract of *Cucumis prophetarum* var. prophetarum fruits on the proliferation of MCF7, MDA-MB-231, HCT-116, A2780, A2780CP, and HepG2. Cell were treated with 0–1000 μg/ml. ^a^ IC_50_: is the concentration that inhibited cell proliferation by 50%. *n* = 8

## Discussion

Bioassay guided fractionation of the methanolic extract of fruits of *Cucumis prophetarum* var. prophetarum led to the identification of ethyl acetate fraction as the most active fraction. The subsequent chromatographic purification of the ethyl acetate fraction resulted in the isolation of five Cucurbitacin compounds. The compounds were characterized based on NMR and mass spectral data.

The ethyl acetate fraction was subjected to column chromatography on silica gel to give three main fractions (I, II, and III). Fraction II afforded pure cucurbitacin B (2) (Fig. 1). Both fractions I and III demonstrated very active cytotoxicity profiles against all cell lines in a concentration-dependent manner, with the IC_50_ value range from 0.15 to 5.9 μg/mL for fraction I and 0.12 to 20.5 μg/mL for fraction III (Table 3). The bioassay guided purification of fractions I and III resulted in the isolation and identification of four cucurbitane-type triterpenes, cucurbitacin E (1), hexanorcucurbitacin D (3), cucurbitacin D (4), and cucurbitacin F 25-O-acetate (5) (Fig. 1). Previously, several cucurbitacin compounds were reported to inhibit the growth of several types of cancers in time-dependent and dose-dependent manners [21]. Cucurbitacin B exhibited inhibitory effects on the proliferation of breast cancer cell lines MDA-MB-231, ZR-75–1, BT474 [22], MDA-MB-453, T47D [22, 23] and MCF-7 [23]; the hepatic carcinoma cell lines BEL-7402 [24] and HepG2 [25]; and the colon cancer cell lines SW480 [26] and HCT-116 [27]. In the same manner, cucurbitacin D and E have shown significant cytotoxicity on the colon cancer cell line HCT-116 [27] and the breast cancer cell line MCF-7 [27, 28]. To the best of our knowledge, cucurbitacin compounds have not been investigated against the human ovarian cancer cell lines A2780 and A2780CP. In addition, this is the first report of screening hexanorcucurbitacin D and cucurbitacin F 25-O-acetate against the six human cancer cell lines used in this study.

Here, we report the inhibitory effects of five cucurbitacin compounds (cucurbitacin E, B, D, F 25-O-acetate, and hexanorcucurbitacin D) obtained from the ethyl acetate fraction on the proliferation of six human cancer cell lines for 48 h. Compounds were re-evaluated against the same cell lines in order to establish a structure activity relationship (SAR) as we describe in the manuscript. Another reason was to ensure that the cytotoxicity activity was consistent with the literature data.

Cell viability was measured in the concentration range of 0 μM to 100 μM for each pure compound. All compounds exhibited antiproliferative activities to the cells in a concentration-dependent manner (Table 5**,** Fig. 4). Among the cucurbitacins that we tested, cucurbitacin B and E showed potent cytotoxicity activities against all six human cancer cell lines at different concentrations, with the IC_50_ value ranging from 0.96 to 16 μM for cucurbitacin B and from 2.1 to 15.9 μM for cucurbitacin E. Meanwhile, cucurbitacin D and Q demonstrated less cytotoxic activity on all six human tumor cell lines than cucurbitacin B and E, with the IC_50_ ranging from 3.4 to 18.4 μM for cucurbitacin F 25-O-acetate and 4 to 26.7 μM for cucurbitacin D. Hexanorcucurbtacin D was the least active of the five cucurbitacins examined, with an IC_50_ ranging from 12.0 to > 100 μM. Interestingly, all cucurbitacin compounds exhibited significant cytotoxic activity against the estrogen-receptor negative human breast cancer cell line (MDA MB-231) compared to the estrogen-receptor. Positive human breast cancer cell line (MCF-7). This significant difference in the biological activities may be related to the status of the estrogen receptor in both cell lines [14]. This was confirmed by using an estrogen- receptor (ER) competitive-binding assay to determine the affinity of cucurbitacin compounds to an estrogen-receptor (alpha and beta). The results confirmed that cucurbitacin compounds possessed very weak affinity toward estrogen receptors and this may explain the significant growth inhibitory effect associated with treatment MDA MB-231.

**Table 5:** Cytotoxic effects of tested compounds

	IC_50_ (μM)					
Isolated compounds	MCF-7	MDA MB 231	A2780	A2780 CP	HepG2	HCT-116
Cucurbitacin E (1)	7.2	2.1	5.4	15.9	3.4	3.4
Cucurbitacin B (2)	16.0	0.96	7.6	14.2	1.7	1.7
Hexanor-Cucurbitacin D (3)	47.9	12.0	> 100	> 100	37.8	30.7
Cucurbitacin D (4)	26.7	4.0	21.6	6.9	5.0	7.6
Cucurbitacin F 25-O-acetate (5)	18.4	3.4	15.8	15.2	10.2	11.2
Dihydrocucurbitacin D (6)	–	> 100	–	–	–	–
Isocucurbitacin D (7)	–	1.0	–	–	–	–
Cucurbitacin E glucoside (8)	–	27.3	–	–	–	–

Inhibitory effects of compounds from the ethyl acetate extract fraction of *Cucumis prophetarum* var. prophetarum fruits on the proliferation of MCF7, MDA-MB-231, HCT-116, A2780, A2780CP, and HepG2. Cell were treated with 0–100 μM. ^a^ IC_50_ is the concentration that inhibited cell proliferation by 50%. *n* = 8

Furthermore, the inhibitory effects of the five cucurbitacin compounds were quite consistent with the trend observed for the activity of fractions I and III, where fraction I demonstrated a more potent cytotoxic activity (IC_50_ value range from 0.15 to 5.9 μg/mL) than fraction III (IC_50_ value range from 0.12 to 20.5 μg/mL). The strong activity of fraction I is probably related to the presence of cucurbitacins B and E, the most active compounds in this study, suggesting that the activity of the two cucurbitacins are synergistic.

In order to establish structure-activity relationships for cytotoxicity against the human breast cancer cell line MDA MB-231, additional cucurbitacin compounds [dihydrocucurbitacin D (6), isocucurbitacin D (7), cucurbitacin E glucoside (8)] were isolated in our laboratory [11] and screened against MDA MB-231 [29].

Our results indicate that the most important structural features for cytotoxicity which are listed below:

(i) The presence of a side chain attached to the four-ringed core structure in the cucurbitacin skeleton. Cucurbitacins B and D, which contain the side chain, exhibited significantly more potent cytotoxic activity (2, 4 IC_50_ = 0.96, 4 μM, respectively) than hexanorcucurbitacin D, without the side chain (3, IC_50_ = 12). This clearly indicated the importance of the side chain since the hydroxyl group at C-16 forms a hydrogen bond with carbonyl group at C-22 on the side chain, leading to the activation of α, β unsaturated ketone [13].
(ii) The presence of an α, β unsaturated ketone in the side chain. Thus, cucurbitacin D, in particular, showed potent cytotoxic activity (4, IC_50_ = 4 μM), while dihydrocucurbitacin D (without an α, β unsaturated ketone in its side chain) showed no activity (6, IC_50_ > 100). This is understandable because α, β unsaturated ketone play important role in nucleophlic attack and consequently alkylation of thiol groups [13].
(iii) The presence of an acetoxyl group at C-25 in the side chain. Cucurbitacin B, which contains this feature, displayed very strong cytotoxic activity (2, IC_50_ = 0.96 μM) compared with cucurbitacin D, which has no an acetoxyl group at C-25 in the side chain (4, IC_50_ = 4 μM). Lipophilicity plays a significant role in transport, absorption and distribution of chemicals in biological systems. Since the presence of acetate group increases lipophilicity, acetylation of C-25 hydroxyl may explain the increase in the cytotoxicity of cucurbitacin B [30].
(iv) The presence of a keto and hydroxyl group on ring A. Thus, cucurbitacin B displayed high activity (2 IC_50_ = 0.96 μM) compared to cucurbitacin F 25-O-acetate, with two hydroxyl groups on ring A (5, IC_50_ = 3.4 μM).
(v) The position of a keto and hydroxyl group on ring A. Isocucurbitacin D, with the keto group at C-2 and the hydroxyl at C-3, demonstrates better activity (7, IC_50_ = 1 μM) than cucurbitacin D, with the keto group at C-3 and the hydroxyl at C-2, (4, IC_50_ = 4 μM).
(vi) The presence of a 2-glucosyl substituent. The cucurbitacin E glycoside (8) has a C-2 glucoside moiety and showed lower activity (4, IC_50_ = 27.3 μM) than cucurbitacin E (1, IC_50_ = 2.1 μM). This is understandable, since the presence of the glucose moiety increased the polarity and the volume of structure, consequently reduces the lipophilicity and transportation through the lipid bilayer of the cell membrane [30, 31]

## Conclusion

*Cucumis prophetarum* var. prophetarum (Cucurbitaceae), called Shari-al-deeb in Arabic, is used in Saudi folk medicine for the treatment of liver disorders. The chemical constituents were defined to determine potential toxicity, mutagenicity, and carcinogenicity. In the present study, bioassay-guided fractionation and purification were used to isolate the cytotoxic compounds of an extract of *Cucumis prophetarum* var. prophetarum frutis. All fractions, sub-fractions, and pure compounds were screened for their cytotoxic activity against six human cancer cell lines. The greatest cytotoxic activity was found to be in the ethyl acetate fraction, resulting in the isolation of five cucurbitacin compounds identified as cucurbitacin E (1), cucurbitacin B (2), hexanorcucurbitacin D (3), cucurbitacin D (4), and cucurbitacin F 25-O-acetate (5). Among the cucurbitacins that were isolated and tested, cucurbitacin B and E showed potent cytotoxicity activities against all six human cancer cell lines at different concentrations. Interestingly, the estrogen-receptor negative human breast cancer cell line (MDA MB-231) was the most sensitive to cucurbitacins B, D, and E, compared to other cell lines. This finding may help us to identify new anticancer compounds against estrogen receptor negative breast cancer.

## Abbreviations

^13^C-NMR: Carbon- Nuclear Magnetic Resonance
^1^H-NMR: Proton-Nuclear magnetic resonance
2D-NMR: Two dimensional- Nuclear Magnetic Resonance
CAM: Ceric Ammonium Molybdate
CDCl_3_: Dueterated chloroform
COSY: Homonuclear correlation spectroscopy
DMEM: Dulbecco’s modified eagle medium
DMSO: Dimethyl sulfoxide
ELISA: Enzyme linked immunosorbent assay
EtOAC: Ethyl acetate
FBS: Fetal bovine serum
HCl: Hydrochloric acid
HMQC: Heteronuclear Multiple-Quantum Correlation
HR-ESIMS: High resolution electron spray ionization mass spectrometry
IC_50_: Half maximal inhibitory concentration
mM: Milli molar
MTT: 3-(4,5-Dimethyl-2-thiazolyl)-2,5-diphenyl-2H-tetrazolium bromide
PBS: Phosphate buffered saline
RPMI: Rosewell Park Memorial Institute
SDS: Sodium dodecyl sulfate
TLC: Thin layer chromatography
TMS: Tetramethyl silane
var.: Variety
μg: Microgram
μL: Microliter
μM: Micromolar
